# Evaluation of Inkjet-Printed Reduced and Functionalized Water-Dispersible Graphene Oxide and Graphene on Polymer Substrate—Application to Printed Temperature Sensors

**DOI:** 10.3390/nano11082025

**Published:** 2021-08-08

**Authors:** Dimitris Barmpakos, Vassiliki Belessi, Rayner Schelwald, Grigoris Kaltsas

**Affiliations:** 1 microSENSES Laboratory, Department of Electrical and Electronics Engineering, University of West Attica, Egaleo, 122 43 Athens, Greece; 2 Institute of Nanoscience and Nanotechnology, National Center for Scientific Research “Demokritos”, P.O. Box 60037, Agia Paraskevi, 153 10 Athens, Greece; 3 Physics Department, University of Patras, 265 04 Patras, Greece; 4 Department of Graphic Design and Visual Communication, Graphic Arts Technology Study Direction, University of West Attica, Egaleo, 122 43 Athens, Greece; 5 Filmetrics, A KLA Company, 82008 Unterhaching, Germany; rschelwald@filmetrics.com

**Keywords:** inkjet-printed sensors, graphene, reduced graphene oxide, flexible temperature sensors, printed temperature sensors

## Abstract

The present work reports on the detailed electro-thermal evaluation of a highly water dispersible, functionalized reduced graphene oxide (*f*-rGO) using inkjet printing technology. Aiming in the development of printed electronic devices, a flexible polyimide substrate was used for the structures’ formation. A direct comparison between the *f*-rGO ink dispersion and a commercial graphene inkjet ink is also presented. Extensive droplet formation analysis was performed in order to evaluate the repeatable and reliable jetting from an inkjet printer under study. Electrical characterization was conducted and the electrical characteristics were assessed under different temperatures, showing that the water dispersion of the *f*-rGO is an excellent candidate for application in printed thermal sensors and microheaters. It was observed that the proposed *f*-rGO ink presents a tenfold increased temperature coefficient of resistance compared to the commercial graphene ink (G). A successful direct interconnection implementation of both materials with commercial Ag-nanoparticle ink lines was also demonstrated, thus allowing the efficient electrical interfacing of the printed structures. The investigated ink can be complementary utilized for developing fully printed devices with various characteristics, all on flexible substrates with cost-effective, few-step processes.

## 1. Introduction

Inkjet-printing for flexible electronics poses several unique advantages over other printing processes in terms of developing direct patterning devices with good feature size and very low material waste [1]. Being a digitally-controlled additive manufacturing process, it offers fast prototyping of various designs without the need of masks or any other photolithographic step. Furthermore, inkjet printing is mainly a non-contact technology, thus it is compatible with a wide variety of substrates [2]. Various materials are compatible with inkjet printing with basic constrains being the viscosity (μ), the surface tension (γ), and the minimum particle size, which alongside nozzle diameter and the jetting speed form an empirical rule of printability, as first described by Ohnesorge [3]. The dimensionless number, Oh (Ohnesorge), correlates these aforementioned parameters via the Re (Reynolds) and We (Weber) numbers; Re is the ratio of inertial to viscous forces, We is the ratio of inertial to surface forces, and Oh expresses the balance between Re and We. Typical values of surface tension for jettable via inkjet materials are in the range of 20 to 40 mN m^−1^, while viscosity is typically in the range of 2 to 50 mPa s. Most inkjet inks exhibit Newtonian behavior, with some examples of inks also showing a degree of viscoelasticity [4]. Although purely aqueous dispersions exhibit characteristics outside the outliers of viscosity and surface tension for printability (water at 20 °C has γ = 72.5 mN m^−1^ and μ = 1 mPa s), in this study, we demonstrate the printability of such dispersions with the appropriate nozzle settings.

There are various studies reported in the literature regarding inkjet-printed sensors on flexible substrate for detecting and measuring relative humidity [5,6,7,8,9,10,11,12,13,14], temperature [9,13,15,16,17,18,19], and various gases [20,21]. Most of these sensors are Ag-based or PEDOT:PSS-based devices [22,23]. Silver nanoparticles, which exhibit high surface to volume ratio, are easy to handle and their sintering strategies, based on Ostwald ripening [1,24], allow for conductivity close to that of bulk silver; it is great for forming conductive geometries, but the costs involved may render the material inappropriate for mass scale production of printed devices. PEDOT:PSS, due to its plasticized nature, exhibits high mechanical flexibility and can easily be processed to increase its electrical conductivity by orders of magnitude, reaching values over 4000 S cm^−1^ [25]; it is also highly transparent. The main drawback of such a conductive polymer is its instability in environmental conditions. Depending on the application, exposure to humidity may cause swelling [26], and decrease its electrical resistance (under high relative humidity) [27].

Graphene is a promising candidate for printed electronics device formation. It is highly conductive, chemically stable and flexible, having a very high mechanical durability. Various methods have been developed to prepare graphene, which can be generally separated into mechanical exfoliation, solution-based and chemically assisted exfoliation, chemical synthesis, epitaxial growth through sublimated SiC surface and pyrolysis of hydrocarbons on metal surfaces [1,28]. Graphene prepared by mechanical exfoliation is considered of the highest quality, but this method has limits for practical applications; solution and chemical exfoliation is considered as the most promising way toward the production of graphene inks, because of the required low-cost raw materials, scalability, and low thermal budget [29]. Graphene dispersions compatible with inkjet printing [29,30,31,32,33,34] can be received either when graphene is directly exfoliated in the organic solvent N-methyl-pyrrolidone (NMP) or when it is stabilized in various organic solvents using ethyl cellulose (EC). Applications of such materials include flexible conductive tracks [29], chemical sensors and TFTs [30], photodetectors [32], and RF circuits [33]. More specifically, inkjet-printed graphene dispersions in cyclohexanone–terpineol as solvents and ethyl cellulose as stabilizer [35] have been used for the development of FETs [36], micro-supercapacitors-energy storage devices [37,38,39,40,41], optical devices and photodetectors [42], sensors [43], and antennas [44].

Graphite oxide, extensively exfoliated in water (graphene oxide, GO) is a non-conductive and oxidized derivative of graphite produced under strong acidic conditions [45]. In order to restore the π-network and thus the electrical conductivity, a chemical reduction process follows, traditionally using a variety of reductive agents such as hydrazine, hydroiodic, or ascorbic acid and more [46] or alongside simultaneous functionalization for additional enhancement of electrical conductivity and dispersibility in cases that rGOs are used in water-based ink technology [47]. An equally important reason to follow this route is that it provides the potentiality for the large scale and low-cost production of rGO.

This work utilizes a one-pot reduced and functionalized GO (*f*-rGO) via sulfonated aromatic diamine, which has been proven to restore an extensive sp^2^ carbon network, thus posing good electrical properties coupled with outstanding water solubility, allowing for use in printed electronics [48]. Furthermore, the present work targets assessing a custom *f*-rGO ink and a commercial graphene ink for compatibility with inkjet printing in order to be used for the implementation of various printed electronic devices. Jetting capabilities of both materials have been studied with an inkjet system, while electrical characterization of both materials (as printed and long term) has been performed. Moreover, electrical interfacing with a commonly utilized Ag-nanoparticle ink has been demonstrated, thus enabling the transition to multi-material device development. The materials have been thermally evaluated for applications in temperature sensing.

## 2. Materials and Methods

Graphite (powder, synthetic, particle size <20 μm) and 2,4-diaminobenzenesulfonic acid (≥98%) were purchased from Merck, KGaA (Darmstadt, Germany). Sulfuric acid (95–97%) and potassium chlorate (purum >99.0%) were purchased also from Merck, KGaA, (Darmstadt, Germany) and nitric acid (65%) from Riedel-de Haen (Munich, Germany). All solvents were of analytical grade and were used as received. A commercial graphene ink (code 793663) with 2.4 wt% solids (graphene and ethyl cellulose) in cyclohexanone and terpineol and a silver nanoparticle ink (code798738), a 30 wt % dispersion in ethylene glycol, were used for inkjet printing. The silver ink was utilized for printing the testing interconnection pads.

### 2.1. Synthesis of f-rGO

Graphite oxide was synthesized by the modified Staudenmaier method [49]. A total of 2 g of powdered graphite were added in an ice-cooled flask containing a mixture of concentrated sulfuric acid (80 mL) and nitric acid (40 mL). Potassium chlorate (40 g) was slowly added to the mixture while stirring and cooling. The reactions were quenched after 18 h by pouring the mixture into distilled water and the product was isolated by centrifugation (13,000 rpm) and washed with water several times until the pH of the supernatant was almost neutral. The sample was then dried at room temperature.

The *f*-rGO was prepared according to [47]. In brief, 100 mg of GO was dispersed in 100 mL of deionized water and stirred for 24 h followed by 30 min ultra-sonication. Afterward, 300 mg of 2,4-diaminobenzenesulfonic acid was added in the mixture and refluxed under magnetic stirring for 2 h. After cooling, the mixture was vacuum filtered through Nylon membrane filters with a 0.45 μm pore size (Whatman). The obtained product was washed extensively with water, ethanol, and acetone. Finally, an appropriate amount of the *f*-rGO was dispersed in deionized water in order to obtain a 3.5 wt% *f*-rGO ink.

### 2.2. Characterization Techniques

A Thetametrisis FR-DEPOSIT (Athens, Greece) drop-on-demand piezoelectric inkjet printer equipped with a Microdrop MD-K-140 nozzle (∅70 μm) and an MD-6020 head controller was used for all the printing processes. The printer was equipped with a hotplate and a controller for nozzle temperature. Droplet formation monitoring was performed via high-speed stroboscopic imaging with a USB3.0 camera (XIMEA, Münster, Germany MQ013MG-E2). First, inkjet compatibility for both inks was evaluated. Prior to printing, the inks were ultrasonicated for 2 min, for resolving agglomerations. The inks were then loaded to vials and an experimental tuning of jetting parameters (voltage and pulse duration, which trigger the piezoelectric element of the printer’s nozzle) was performed.

The 125 μm-thick polyimide substrate (DuPont Kapton HN, Wilmington, DE, USA) was treated with 1 M NaOH for 7 min for increasing wettability; afterward, the substrate was successively rinsed with acetone, deionized water, and isopropyl alcohol. The samples were loaded to the printer hotplate, which was kept at 34 °C at all printing sessions, in order to enhance the process repeatability and slightly assist with solvent evaporation. Moreover, this technique prevents the samples from absorbing environmental humidity during printing.

To acquire the 3D topographies of the printed samples, a Filmetrics Profilm3D (Unterhaching, Germany) white light interferometer-phase shift interferometer was utilized. The device incorporates a broadband white-light source and Mirau interference objective lenses. Information about the sample coloring was also acquired as a function of topographic height through Filmetrics TotalFocus™. A FLIR A655SC IR, (Wilsonville, OR, USA) camera was used for monitoring the samples’ temperature during all temperature-related experiments.

The test designs were loaded in the printer software, where the user can adjust the distance per pixel (droplet), which determines the droplet overlapping in the actual printed geometry. Given the fact that droplets have a diameter of approximately 80 μm mid-flight, a distance of droplet centers in the range of 60–70 μm in both axes yielded the optimal experimental results, depending on the ink. It should be noted that the G and *f*-rGO inks exhibited a different spreading behavior as a direct implication of different solvent use; graphene ink contains cyclohexanone and terpineol while *f*-rGO ink is purely water dispersed. Therefore, the inter-droplet spacing was adjusted accordingly, after observing the printed structures via optical microscopy. The design consisted of 500 × 8 pixels parallel lines as well as a 500 × 500 pixel square.

## 3. Results-Discussion

**Figure 1:**
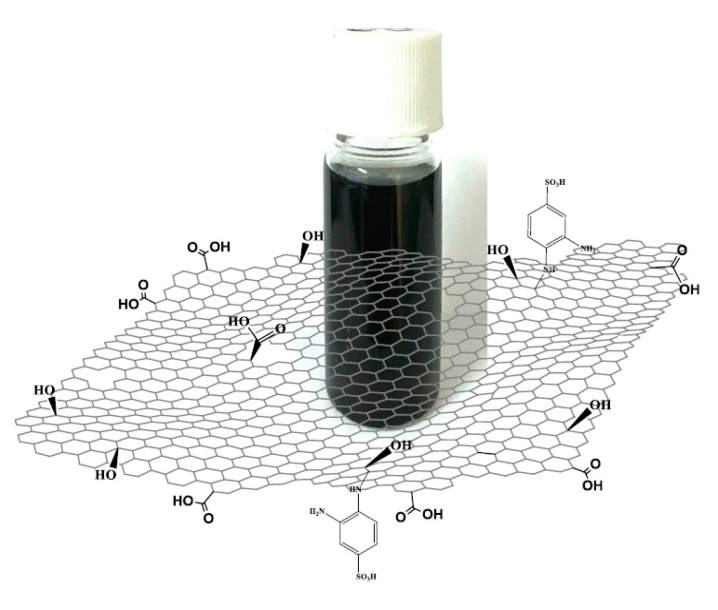
Schematic representation of the *f*-rGO ink.

The *f*-rGO is a highly dispersible material in various solvents (water and organic) without the addition of surfactants or other stabilizers. This is due to the presence of sulfonated aromatic diamines, which are covalent bonded in the rGO sheets as derived from the characterization of similar samples with FTIR spectroscopy and x-ray photoelectron spectroscopy in our previous research work [48]. The same methods confirm the presence of carboxylic and carbonyl groups in the surface of the rGO nanosheets. Practically, the reduction of GO with the 2,4-diaminobenzenesulfonic acid leads to a highly reduced and hydrophilic graphene derivative [47,48] (Figure 1). In addition, the 3.5 wt% *f*-rGO water dispersion remains stable for at least one month and is ready to be used after shaking of a few seconds. The commercial graphene ink is produced by the dispersion of graphene and ethyl cellulose powder in cyclohexanone and terpineol [29].

### 3.1. Droplet Formation—Printability

**Figure 2:**
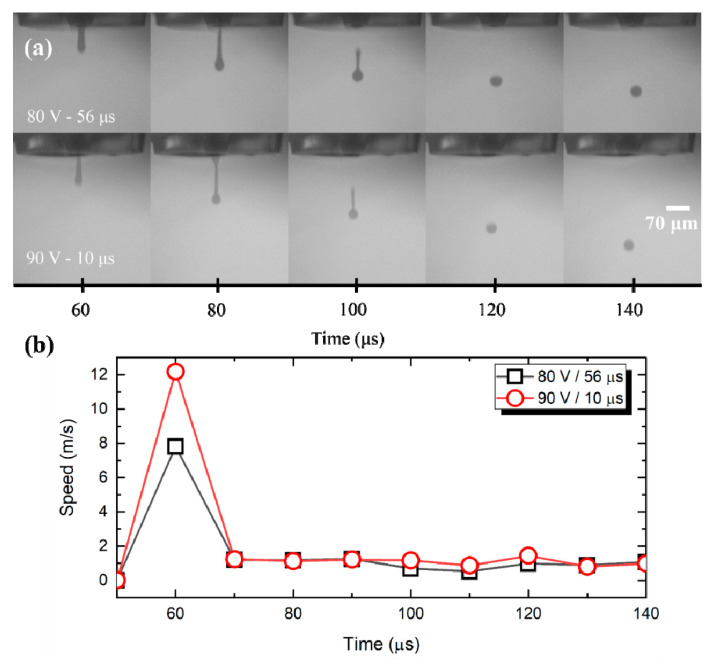
Droplet formation of graphene ink. (**a**) White labeled text indicates the inkjet settings, while the X axis corresponds to the delay after firing the piezoelectric element and the image acquisition; (**b**) calculated speed for graphene ink.

G ink jetting is relatively straightforward because the ink is commercially available and inkjet-printer compatible. It was observed that various combinations of voltage and pulse duration of driving pulses are capable of accurately producing repeatable results. As can be seen in Figure 2a, by increasing pulse duration, more ink was ejected per droplet, considering that the droplet shading (photographs are in grayscale) was more intense and the diameter was larger. On the other hand, as expected, slightly higher voltages with lower duration resulted in smaller droplets with higher speed (Figure 2b); for the following experiments, an 80 V-56 μs combination was selected.

**Figure 3:**
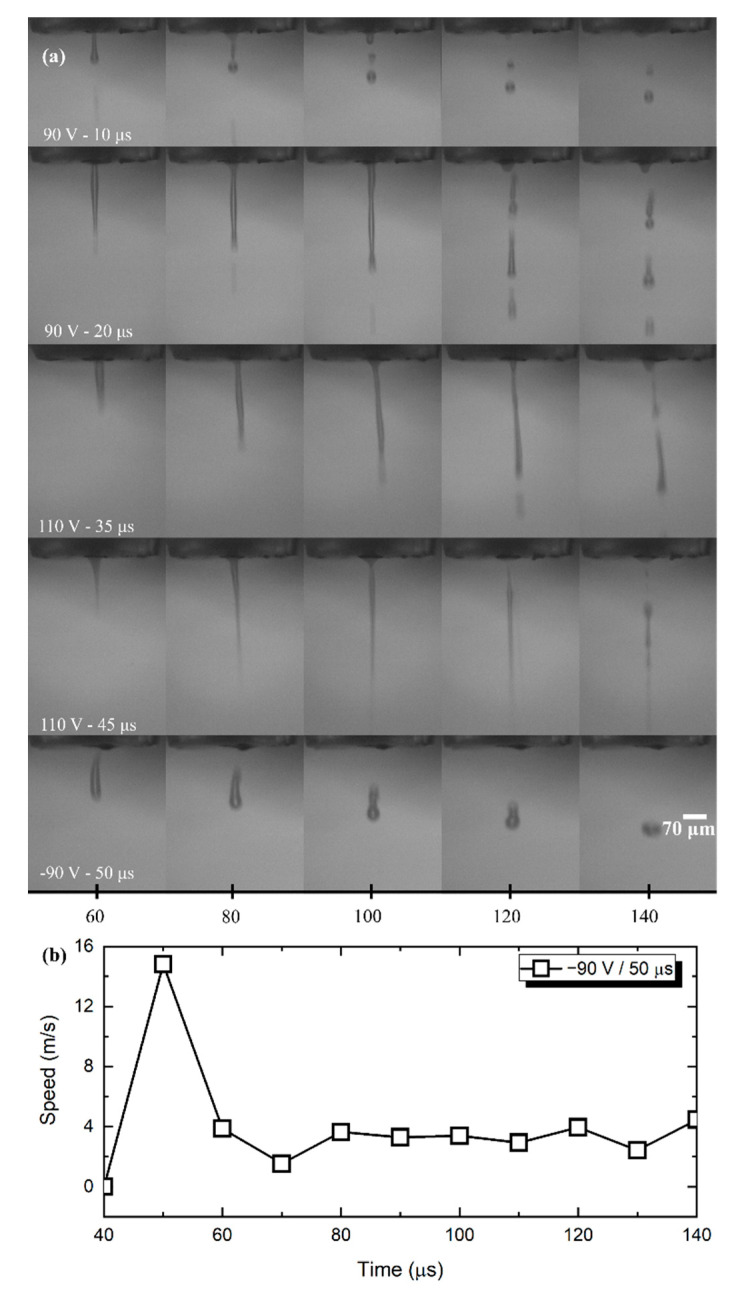
Droplet formation of *f*-rGO ink. (**a**) Lower positive voltages resulted in early jetting breakup; higher positive voltages resulted in transferring an oscillation mode to the jetted line before breakup of the tail to droplets of equal size. By reversing the voltage, headroom for adjusting pulse duration was gained. White labeled text indicates the inkjet settings, while the X axis corresponds to the delay after firing the piezoelectric element and the image acquisition; (**b**) calculated speed for −90 V/50 μs pulse.

The *f*-rGO custom ink is water-based, therefore it exhibits viscosity and surface tension similar to that of plain water. Although it is possible to jet water-based inks from inkjet printers, there exists a tighter area where printing conditions are met, in contrast to inks with more viscous solvents. As presented in Figure 3a, jetting break-up occurs for lower voltages and pulse duration (<80 V and <15 μs, respectively), which results in satellite droplets; higher voltages—pulse durations extrude excessive ink. This jet perturbation is known to be caused by the superposition of Rayleigh–Plateau-unstable modes [50] triggered by the specific waveform.

By reversing the pulse polarity and keeping the voltage at −90 V for 50 μs, repeatable jetting occurred, therefore the final settings used for the following experiments was kept to the above-mentioned values. The voltage polarity influence in droplet formation is correlated to the structural details of the printhead alongside the pressure wave propagation-reflection inside the nozzle. Piezoelectric element placement and mode of operation (squeeze, bend and so on) are responsible for the completely different results when pulses of the same amplitude with opposite signs are driving the nozzle, as indicated in Figure 3a. Therefore, a negative voltage of higher duration ejects enough ink with sufficient velocity (Figure 3b) to detach from the main meniscus. It should be noted that no follow-up quenching pulse has been implemented; typical inkjet driving waveforms include a driving and a quenching pulse of opposing signs, for assisting in residual oscillation damping inside the printhead after droplet ejection [4,51]. We followed the most essential route to keep the driving electronics as simple as possible, validating that a single pulse is sufficient for printing both materials.

### 3.2. Printed Geometry Analysis

**Figure 4:**
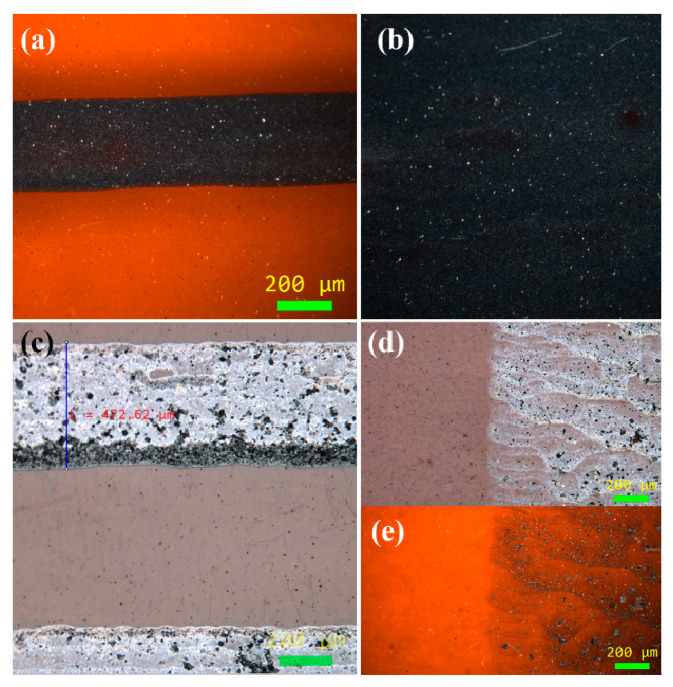
Printed graphene line (**a**) and area (**b**); printed *f*-rGO line (**c**) and area (**d**), dark filter; (**e**), bright filter.

Next to the droplet formation analysis, printing of the test pattern took place. Specifically, a line of 500 × 8 pixels and an area of 500 × 500 pixels with a droplet overlap of 65 μm in both axes were formed in single-pass printing, followed by a sintering at 240 °C for 1 h. It was observed that uniform, continuous lines were formed. Some areas (where the lines overlapped) exhibited edges that correspond to each printed line due to slow printing speed; moreover, the ink’s solvent that was deposited as first line needs some seconds to start drying, leading to printing of the successive line onto a semi-dry area, thus height is partially added. Figure 4a,c presents the graphene and *f*-rGO inkjet-printed lines, respectively. The corresponding printed areas are shown in Figure 4b,d,e. The *f*-rGO photographs were taken with both the dark (4d) and bright filter (4e).

**Figure 5:**
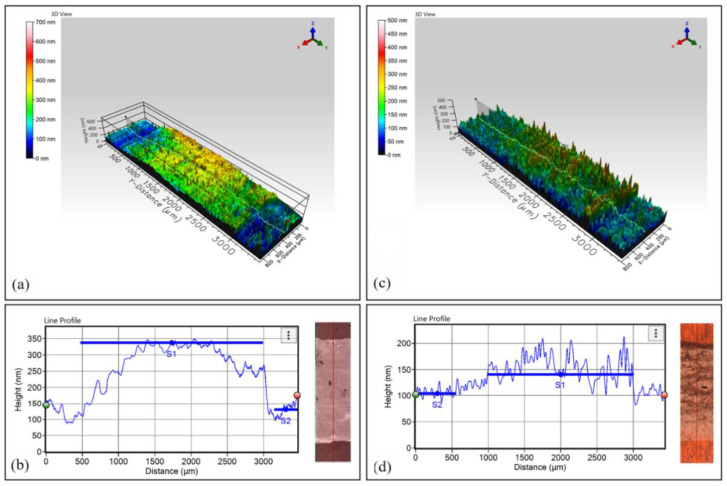
3D measurement for graphene (**a**) and *f*-rGO (**c**) and height profile measurements for graphene (**b**) and *f*-rGO (**d**). The insets show a top view of the areas for which the 2D height extraction was performed, obtained by Filmetrics TotalFocus™. Linewidth is 800 µm (**b**,**d**).

The 3D topography results highlight the impact of the solvent utilized and the effect of droplet overlapping: for graphene ink, whose solvent is cyclohexanone and terpineol, evaporation was relatively uniform across the sample line, therefore a coffee ring distinguishing pattern was absent in the line edges. Figure 5a,b illustrates the 3D cross-sectional profile of a graphene and a *f*-rGO line, respectively. The graphene line height was 149 nm ± 21 nm. Water-based *f*-rGO geometries exhibited a typical coffee ring pattern (visible in Figure 8a). This was attributed to faster evaporation on the line edges (assisted by the drying temperature), which induces a flow that moves the nanostructures from the droplet center toward the edges [52]. On the other hand, the absence of such effect on the graphene lines can be explained by the boiling point mismatch of terpineol and cyclohexanone; it has been observed that solvents with different boiling points can, under the appropriate drying temperature, completely suppress this effect [53]. The *f*-rGO line height was 50 ± 31 nm.

### 3.3. Electrical Characterization

**Figure 6:**
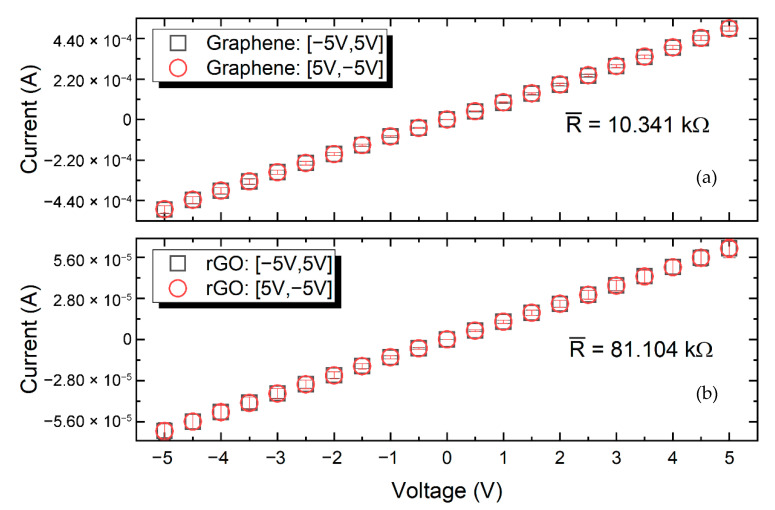
IV curve of inkjet-printed graphene (**a**) and *f*-rGO (**b**) after annealing at 240 °C.

A series of several printed samples (designed as 5 mm × 0.5 mm) of each ink were characterized using a custom prober setup connected to a Keithley 2612 source-meter, in order to extract the corresponding electrical resistance. Figure 6 presents the results for both inks, where the errors bars indicate the related deviations among the evaluated sample-set. In all cases, voltage was applied in the range of −5 to 5 V and the corresponding current was measured. The duration of each volage step was 2 s. The inter-sample resistance variation was below 5%. Graphene and *f*-rGO samples exhibited a mean resistance of 10.34 kΩ and 81.10 kΩ, respectively. The samples’ actual printed width and height differed, as explained in the previous paragraph, which had an impact on the extracted absolute electrical resistance value. Nevertheless, the sizes were comparable and these measurements should be taken into account to grasp the magnitude of each ink’s resistance; on the other hand, both inks showed good compatibility with the inkjet, thus enabling the development of multi-material graphene-based devices. As has already been presented in the literature, printed materials with different resistance values can substitute traditional resistors of various values [54]; by using materials with different resistivity, it is possible to achieve specific resistance values with enhanced precision. The long-term evaluation revealed that both materials exhibited a small variance in resistance one month after printing, but stabilized afterward.

To extract the mean resistivity for each ink, the topological data alongside the electrical resistance measurements were combined using Equation (1).

ρ = R × A/L(1)

where ρ is the resistivity; A is the cross-section area; R is the measured resistance; and L is the distance between the probes. The calculated resistivities for graphene and *f*-rGO inks were ρ_G_ = 0.0247 Ω∙cm and ρ*_f_*_-rGO_ = 0.0649 Ω∙cm.

### 3.4. Contact with Ag-Nanoparticle Ink

**Figure 7:**
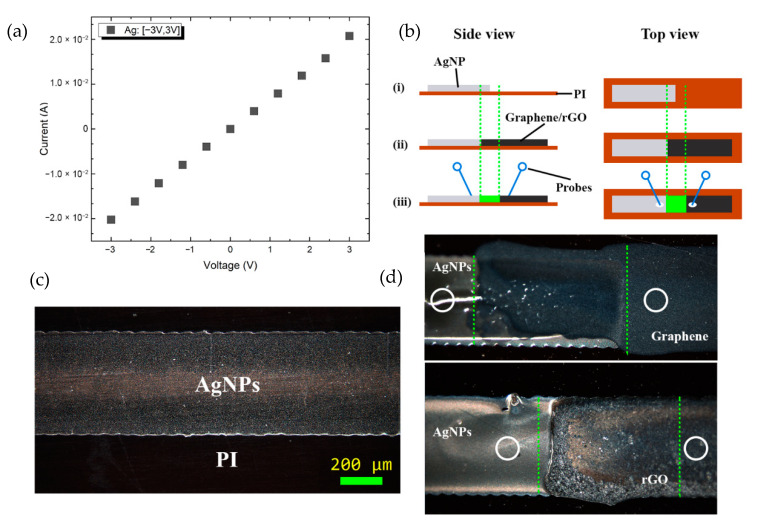
IV curve of printed AgNP line (**a**); printed AgNP line optical microscope image (**b**); experimental process for the contact between G/*f*-rGO ink and AgNP: printed AgNP (i), printed graphene and *f*-rGO inks with an overlap area depicted in green (ii), probes position for measuring electrical resistance (depicted in white) (iii) (**c**); *f*-Rgo–AgNP contact optical microscope images. Green lines indicate the total overlap area, while white circles indicate the approximate probe position for measurements (**d**).

A major issue in printed electronic devices is the interconnection between the various printed areas as well as the connection with the contact pads aiming at achieving reliable communication with external devices. In order to address this issue, the direct electrical contact with an Ag-based commercial ink was evaluated for both materials under study. For the characterization of the presented inks’ interfacing with other commonly utilized printable materials, a silver nanoparticle ink (Sigma Aldrich 798738) was printed onto Kapton and sintered at 140 °C for 2 h, forming 450 μm-wide lines (Figure 7b,(ci)), similar to the ones printed with G and *f*-rGO. The corresponding I–V curve (Figure 7a) indicates that good electrical conductance was exhibited as expected. Next, G and *f*-rGO lines were printed to overlap the AgNP line for approximately 350 μm (Figure 7(cii),d).

**Figure 8:**
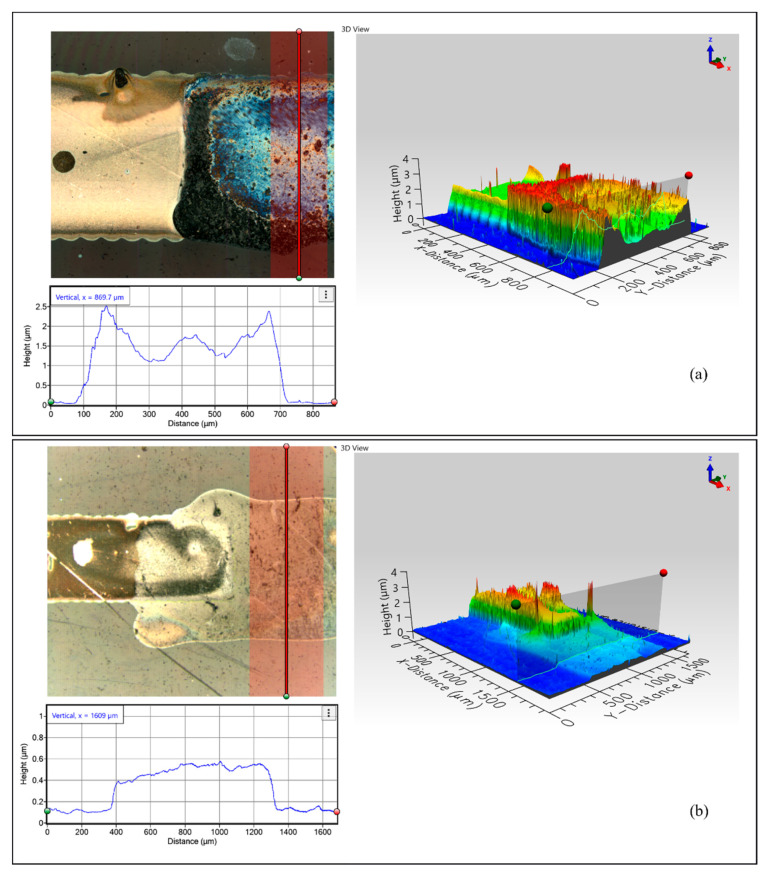
3D measurement for AgNP–*f*-rGO (**a**) and AgNP–G (**b**) interconnections. The insets show height profiles of the *f*-rGO (**a**) and graphene (**b**) regions as indicated in the 2D top view obtained by TotalFocus™.

The 3D topographical measurements for the contacts between AgNPs and *f*-rGO/G confirm that the AgNP line was overcoated in both cases, thus leading to a successful electrical interface (Figure 8). More specifically, regarding AgNP-(*f*-rGO) contact, the second ink was contained between the boundaries defined by the AgNP printed line, leading to a uniform line width throughout the sample, in contrast to the AgNP-G contact, where the G ink was spread over the AgNP line. It can be observed from the 3D interferometry images that AgNPs as a printing substrate can provide adequate wettability for overprinting. Nevertheless, as observed by the electrical measurements, in both cases, electrical interface was achieved.

**Figure 9:**
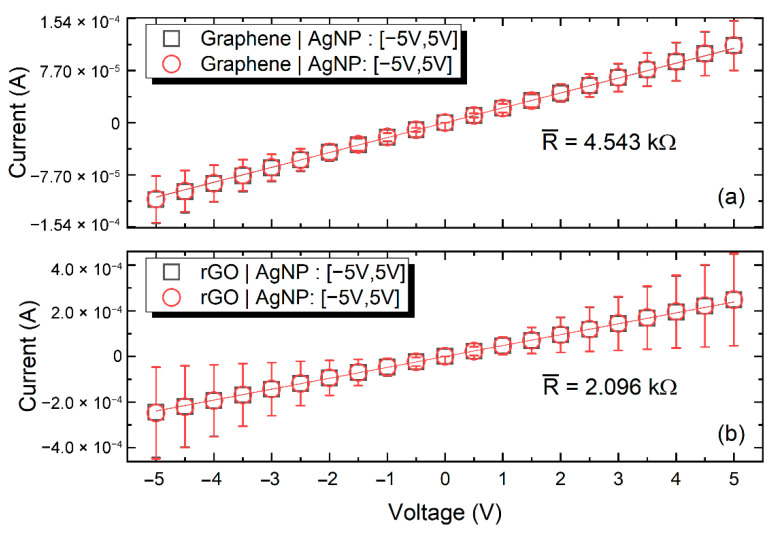
Graphene-AgNP junction IV curve (**a**); *f*-rGO-AgNP junction IV curve (**b**).

To assess the electrical interface of printed G and *f*-rGO with inkjet-printed AgNP lines, two needle probes were utilized to engage close to the junction (Figure 7ciii). For the extraction of the resistance, a voltage sweep was performed in the rage between −5 to 5 V, followed by the measurement of the corresponding current (Figure 9). Measurements for several test junctions for each ink revealed that the graphene–AgNP interface had a mean resistance of 4.54 kΩ while (*f*-rGO)–AgNP interface had a mean resistance of 2.09 kΩ. Nevertheless, for both inks, it is evident that standard deviation is high, while both junctions exhibited an ohmic behavior. The specific experiment demonstrates the efficient coupling of both inks under study with Ag-based printed lines, thus rendering the electric interfacing of G and *f*-rGO printed devices feasible.

The extracted resistance values that are reported above are indicative, since in each case, the addition of an extra contact resistance value should be taken into consideration. It should be noted that these measurements were performed for a preliminary evaluation of the electrical interface of printed AgNP and G/*f*-rGO; ongoing work includes a throughout investigation of contact resistance extraction alongside topology characterization of the junctions for a detailed characterization of the material interface. Nevertheless, AgNP-(*f*-rGO) lower contact resistance could be attributed to the absence of additional organic materials, which is not the case for graphene ink; ethyl cellulose could cause partial insulation between the sintered Ag nanoparticles and graphene sheets, therefore contributing in an increased contact resistance between the two printed structures.

### 3.5. Resistance—Temperature Behavior

**Figure 10:**
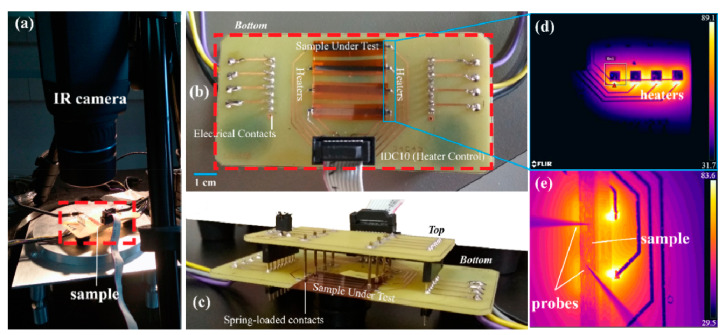
Experimental setup (**a**–**c**); heating of thermistors with 40 mA each under IR monitoring (**d**); top view from the IR camera with active heaters under a sample and the probes engaged for measurement (**e**).

According to the literature, both G and *f*-rGO exhibited a negative temperature coefficient of resistance (TCR), therefore, the materials’ electrical conductivity rises with temperature. To measure the response of the printed samples to various heating conditions, an experimental setup was developed, which allows for selective heating of the substrate’s back plane, where the samples were printed onto (Figure 10). This approach allows for assessing the temperature–resistance relationship in real-world applications, where a sensor implemented in the setup could monitor the temperature under the substrate, thus permitting the temperature measurement at the outer surface of devices developed on flexible substrates like polyimide. With this method, the printed structures are protected from direct exposure to the sensing environment. The setup consists of two rows of Vishay PTS060301B100RP100 thermistors that act as both heaters and temperature sensors. The engagement and mechanical fixture for the samples under test are performed via spring loaded contacts and rows of 2.54 mm pin headers, respectively. The samples can be placed in either orientation, perpendicular or in parallel, to the IDC10 connector. That way, either heating of the entire sample occurs for extracting the resistance–temperature relationship, or one side of the sample is heated while the other remains at room temperature for performing differential temperature evaluation in order to determine various temperature-related parameters (e.g., Seebeck coefficient).

Figure 10a presents the measurement setup with the sample mounted onto the custom PCB housing (in dashed box) and the IR camera monitoring the thermal distribution, while the needle probes were engaged for electrical measurements. The PCB housing is presented in Figure 10b,c; Figure 10d presents a thermal image during the operation of all four heaters on the left side of the setup at 40 mA each; Figure 10e illustrates a thermal image of a 40 mm × 0.5 mm printed graphene line on the Kapton substrate placed on top of the heaters. The engaged probes are also visible in the same image. It has been observed that throughout the whole range of applied heating currents, a practically uniform thermal profile was induced via the substrate to the printed geometries. Two types of printed lines (graphene and *f*-rGO) with the same dimensions (15 mm × 5 mm) were placed in the experimental setup of Figure 10, in order to evaluate the corresponding thermal responses. Both steady state and transient thermal behavior were studied for the two printed materials under evaluation.

**Figure 11:**
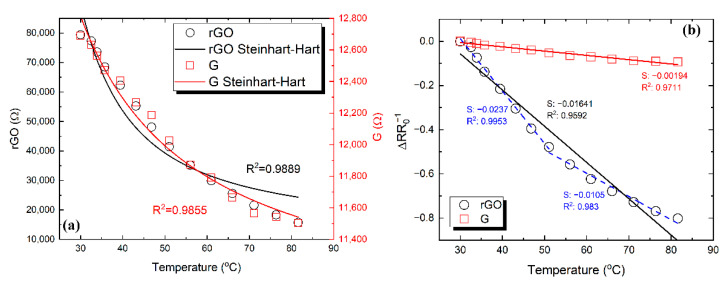
Temperature–resistance relationship for the graphene and *f*-rGO printed samples. R(T) curves for both samples alongside fitting using the Steinhart–Hart equation (**a**); relative resistance change with temperature and linear fitting lines (**b**).

In the first experiment presented in Figure 11a, successive heating in the range of 30–82 °C was applied while the resistance was continuously monitored. The standard thermistor equation, represented by the Steinhart–Hart relationship (Equation (2)) was used to fit the measurement data. It was observed that by using the Steinhart–Hart equation (Figure 11a), the sensors’ response can be described adequately (*R*^2^ = 0.9889 for *f*-rGO and *R*^2^ = 0.9855 for graphene).

1/T = A∙R + B∙ln(R) + C∙ln(R)^3^ (2)

The A, B, C parameters from fitting with Steinhart–Hart are presented in Table 1.

Each samples’ TCR can be extracted from the corresponding results, as indicated in Figure 11b, where the normalized resistance change is presented as a function of temperature. Graphene was greatly outperformed by *f*-rGO in terms of response to temperature variations, exhibiting a negative TCR of −1.94 × 10^−3^ °C^−1^, with *f*-rGO presenting −1.64 × 10^−2^ °C^−1^. For more precise data approximation, *f*-rGO measurements can be fitted in two linear regions (first region 30–48 °C, second region 55–83 °C), as indicated in Figure 11b. In this case, the extracted TCR values were −2.37 × 10^−2^ °C^−1^ and −1.05 × 10^−2^ °C^−1^, respectively.

**Figure 12:**
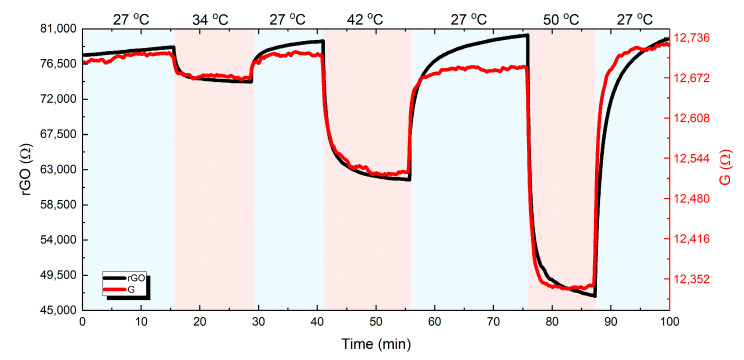
Graphene and *f*-rGO transient response for three heating steps.

In the next stage, the transient thermal response of both graphene and *f*-rGO was assessed. The two samples underwent several heating steps in successively higher temperatures up to 50 °C. After each heating step, the samples were allowed to reach the reference room temperature (27 °C). The results are presented in Figure 12. Recovery to initial resistance was observed for both samples up to 50 °C, but full response and recovery time may require several minutes for higher temperatures.

**Figure 13:**
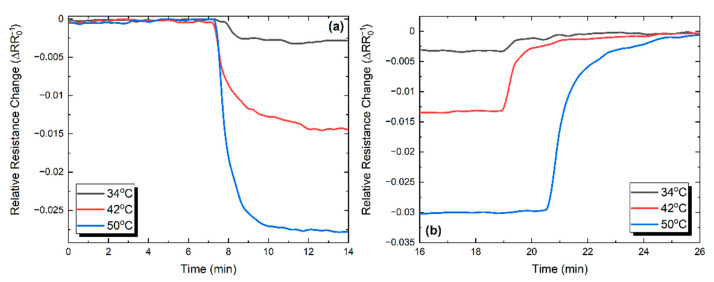
Normalized response (**a**) and recovery (**b**) behavior for three thermal pulses for the graphene sample.

The detailed graphene response and recovery behavior to three successive thermal pulses (34, 42, and 50 °C) is illustrated in Figure 13a,b. In order to quantify the obtained results, response and recovery time is defined according to the terms rise and fall time, as the time taken by the signal to change between 10% and 90% with respect to the reference value, which is the resistance value at room temperature. Following this definition, the mean response and recovery times for graphene were calculated as 2.47 and 3.19 min, respectively.

**Figure 14:**
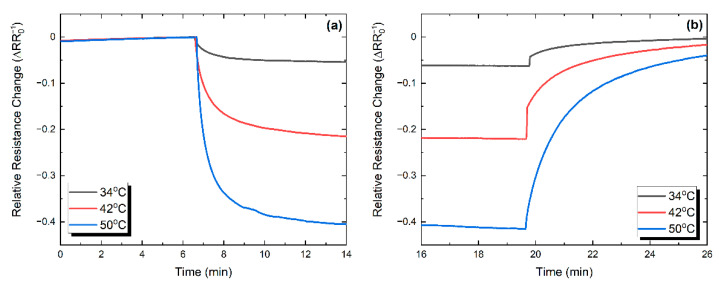
Normalized response (**a**) and recovery (**b**) behavior for three thermal pulses for the *f*-rGO sample.

The corresponding transient thermal response for the *f*-rGO case is presented in Figure 14a,b. Similarly, the calculated response and recovery times for the *f*-rGO samples in each thermal pulse were calculated as 2.94 and 5.28 min, respectively.

In both samples and all the evaluated temperatures, the response phase was considerable faster than the recovery as expected, since the temperature increase was caused by the direct power application, while the recovery was dominated by natural convection. Comparing the two materials, graphene exhibited a slightly faster response and recovery time. This can be attributed to the differences in the combination of thermal conductivity and thermal capacitance of the two materials, but there are no solid scientific results yet to support the specific assumption. Future work will include both geometry optimization and detailed study of the response and recovery time in the full working range and the extraction of the main thermal parameters of each material.

The vision of this work is the development of fully printed graphene-based electronics on flexible substrates, which requires the design and implementation of various interfaces between printed materials alongside electrical and topographical characterization, in order to describe in detail, the appropriate combinations. In addition, the group is actively working on the evaluation of the herein presented materials for printed micro-heater applications, keeping in mind that the exhibited TCRs alongside the measured electrical resistance strongly indicates that these materials are suitable candidates for such application.

## 4. Conclusions

This work studied various aspects for the development of graphene-based printed electronics on flexible substrates. A commercial graphene and ethyl cellulose ink in cyclohexanone–terpineol and an *f*-rGO water-based ink were evaluated for printability and compatibility with inkjet printing. Electrical, optical, and 3D topographical measurements were conducted for characterization of the printed structures; both inks successfully formed continuous, electrically conductive tracks on the Kapton substrate. Afterward, an initial approach on using these inks for the development of multi-material inkjet-printed electronics was presented, where electrical interface between both graphene-based materials with a commercial silver nanoparticle-based conductive ink was investigated. This type of ink is commonly used for the development of electrodes on various devices such as sensors and printed FETs; the results highlighted that such fully printed, multi-layer devices based on graphene can be developed, with electrical contacts between the printed materials adding a considerable ohmic resistance. Finally, an application for temperature sensing was designed and implemented with a custom setup for the measurement of electrical response in the range of 30–82 °C. Both materials exhibited a negative TCR, while the custom *f*-rGO ink outperformed graphene ink in terms of TCR value by approximately an order of magnitude. The two materials’ response and recovery time were found to be similar in the order of some minutes, with graphene exhibiting a mean faster recovery and response time.

## Figures and Tables

**Table 1:** Fitting parameters for both temperature sensors.

	A	B	C
*f*-rGO	0.42093	−0.06344	0.00023
Graphene	60.64707	−9.77378	0.03762

## Data Availability

The data presented in this study are available on request from the corresponding author.
